# Lessons from a Free Health Fair Organized by Students for the Underserved Population in the Post-Pandemic Era

**DOI:** 10.1007/s10903-025-01712-y

**Published:** 2025-06-09

**Authors:** Pinyu Chen, Walter Duy, Amos Wu, Alexandra Jennings, Monica Brown, Melanee Mills, Michael Lischke

**Affiliations:** 1 Wake Forest University School of Medicine, Winston-Salem, USA; 2 Boston Medical Center, Boston, USA

**Keywords:** Free Community Health Fairs, Medical Students, Health Equity

## Abstract

Winston-Salem, North Carolina is one of the poorest cities in the state, with a city-wide poverty rate of 20.6%. Poverty rates in the neighborhoods located to the east and south of Wake Forest University School of Medicine continue to rise over the years. Many residents are uninsured or underinsured and don’t have a primary care provider. Share the Health Fair (STHF) is an annual, one-day student run event that provides free health screenings to the Winston-Salem area community, regardless of income, insurance, or immigration status. STHF was paused for 2 years because of the pandemic and restarted in 2022. Screenings offered include core health screenings, STI, glucose and cholesterol tests, dermatology, pulmonology, vision, pap smears, and dental. Survey data from STHF attendees in 2022 and 2023 were collected. The lack of healthcare maintenance exacerbated by the pandemic has led to an increased attendance at STHF. The demographics changed since before the pandemic– increasing to 90% Hispanic or Latino attendees in 2023, with 78% of the attendees being uninsured and 82% making less than $30,000 a year. Dental and vision screenings remain the most sought-after free services among the attendees.

## Introduction

Share the Health Fair (STHF) was found in 1998, with the mission to provide free health screenings and services to the Winston-Salem community and surroundings, primarily to those who may not otherwise have adequate access to these services. For many of the fair’s attendees, STHF is the only time in a year when they have access to any form of healthcare. The fair is open to the community, regardless of immigration and insurance status and without requiring any documentation. STHF is an entirely medical student-run effort at the Wake Forest University School of Medicine (WFUSM), in partnership with the Downtown Health Plaza (DHP) in downtown Winston-Salem and Northwest Area Health Education Center (NW AHEC). This fair provides a unique opportunity for medical students to interact with and learn from the underserved population in Winston-Salem while gaining hands-on clinical experiences. Unfortunately, because of the COVID-19 pandemic, the fair was canceled for two years from 2020 to 2021 and reinstated in 2022.

Winston-Salem is located in Forsyth County and faces a lot of healthcare disparities and city-wide poverty rates at 20.6%, predominately located in the neighborhoods to the east and south of where the medical school is located [1–3]. In 2018, only the downtown neighborhood had an improved poverty rate, while all other areas have worsened from low to high poverty designations [3]. The median household income is $62,319, with an unemployment rate of 42.2% and 9% of the population living without health care coverage. The racial breakdown of the county from highest to lowest percentage are:: white, black or African American, Hispanic or Latino, and Asian population, with 215,000, 95,000, 55,000, and 9,000 individuals, respectively [4]. The average death rate in Forsyth County (801.6) during 2016–2020 was higher than the overall state average (793.7) [1]. In 2018–2020, the life expectancy (defined as the average number of additional years that someone at a given age would be expected to live if current mortality conditions remain constant) for white population aged 50–54 was 31.9 years and 29.2 years for the black population [5]. For every age group listed, the black population consistently had a lower life expectancy compared to other races and made up ~ 50% of the high poverty neighborhoods [2, 3]. STHF exists to help address these health discrepancies by minimizing barriers to care, improving social determinants of health, and increasing awareness of preventative measures to avoid common chronic diseases.

All services are offered free of charge to every fair attendee, and attendees can pick and choose which screenings to receive. Services offered at STHF include core health screenings (including weight, body mass index, blood pressure), and blood tests (glucose, cholesterol, sexually transmitted infections). Specialty-specific screenings include dermatology (skin cancer), pulmonology (pulmonary function testing, sleep apnea), ophthalmology (retina, glaucoma, visual acuity, visual fields), dental (cavities, refresh procedures), and physical/occupational therapy (gait, balance, walking). Other free services include pap smears, bone density scans, and flu vaccinations. As fair attendees complete the screenings, results are immediately provided and documented in a pamphlet that attendees can take home. The pamphlet is filled with educational materials to teach them what their results mean and preventive health strategies to stay healthy. Attendees with abnormal results are seen by attendings who staff each screening station that can provide further evaluation. Attendees who require follow-up care, are referred to free local community organizations and clinics to receive formal care.

Given the health disparities that exist within nearly every community, it is important for medical students to develop a community-centered approach to healthcare and sensitivity towards patients who face significant barriers to care [6]. This increased awareness produces more holistic physicians that are better prepared to serve the community. Interdisciplinary, medical student-led health fairs have been used as a model for delivering care to the underserved community for years with successful impact and sustainability over the years [6–9]. The purpose of this project is to determine the healthcare needs of the underserved population in Winston-Salem and how to improve future fairs to better address the post-pandemic disparities identified during the 2022 and 2023 Share the Health Fairs.

## Methods

### Advertisement

All advertisement for STHF were created and distributed in Spanish and English. Paper fliers with the date, time, location, and services available at the fair were distributed to local churches, gyms, community centers, libraries, and shelters within Winston-Salem. They were also distributed to DHP and local free clinics that referred patients to STHF for services that they could not offer. STHF has a Facebook page where multiple posts were made with information related to the fair and the specific services offered. Advertisement spaces were made in Que Pasa (a Spanish language media company in North Carolina) as well as on online community calendars to local news stations. There were no inclusion or exclusion criteria for who could attend the fair. STHF has a dedicated website hosted on WFUSM page with event and contact information (https://school.wakehealth.edu/STHF and https://school.wakehealth.edu/STHFspanish).

### Registration and Survey Collection

STHF attendees had the option to pre-register prior to the fair to expedite check-in on the day of. Attendees could also walk in and register on the day of the fair. Every adult attendee was asked to individually fill out the survey with basic demographic information and answer some questions about current health maintenance and barriers to care. Attendees were not asked to provide any legal documentation to attend the fair. Once registered and checked-in, attendees were each given a pamphlet that outlines the screenings stations available with a place for results to be written down, and preventative education pertinent to the station. Participants could pick and choose whichever screening stations they desired, in no particular order. After attendees had completed their desired screenings, they were asked to voluntarily fill out a check-out survey about their experiences at their fair and feedback.

### Data Analysis

This study was approved by the Wake Forest University Health Sciences Institutional Review Board (IRB00113637). The check-in and check-out survey data were collected through Microsoft Forms, and the data were collected and analyzed through Excel for each respective year.

## Results

### Sample Size

In 2022, 383 adults filled out the check-in survey and 185 adults filled out the check-out survey. In 2023, 347 adults filled out the check-in survey and 83 adults filled out the check-out survey.

### Demographic Information

In 2022, a total of 383 adults participants attended STHF, with 72% being female, 77% identifying as Hispanic or Latino, and 57% categorizing their race as other (Table 1). The median age was 41–50 years old, with 80% of the participants living in households earning less than $30,000 total annually (Table 2). When stratified for ethnicity, there were proportionally more Hispanic or Latino attendees in the age groups less than 50 years old. Of the attendees, 78% did not have health insurance and 65% did not have a primary care provider (PCP). Of those with insurance, 84% have a PCP. Out of the individuals without insurance, 21% had a PCP. In the past year, 47% of the patients had delayed care that they felt they needed, with the highest cited reason as not being able to see the provider they wanted to (Table 3). In 2022, 10% of the attendees had been to STHF before.

In 2023, a total of 347 adults participants attended STHF, with 65% being female, 90% identifying as Hispanic or Latino, and 56% categorizing their race as other (Table 1). The median age was 41–50 years old, with 82% of the participants living in households earning less than $30,000 total annually (Table 2). When stratified for ethnicity, the proportion of ages did not change significantly. Of the attendees, 84% did not have health insurance and 82% did not have a PCP. Of those with insurance, 60% have a PCP. Out of the individuals without insurance, 9% had a PCP. In the past year, 32% of the patients had delayed care that they felt they needed, with the highest cited reason being the cost of healthcare (Table 3). In 2023, 16% of the attendees had been to STHF before.

**Table 1:** Demographics of STHF Attendees in 2022 and 2023

Year	Total	Sex		Ethnicity		Race			
		Female	Male	Hispanic or Latino	Non-Hispanic or Latino	White	Black or African American	Asian	Other
2022	383	72%	28%	77%	23%	22%	17%	3%	57%
2023	347	65%	34%	90%	10%	25%	13%	1%	56%

**Table 2:** Household Income and Access to Health Care of STHF Attendees in 2022 and 2023

Year	Total Household Income (per 1000s)				Insurance Status				No PCP
	< 15	15–30	30–45	> 45	None	Medicaid	Medicare	Private	
2022	209	97	41	25	78%	6%	8%	8%	65%
2023	105	46	25	8	84%	6%	3%	7%	82%

**Table 3:** Cited Reasons for Delaying Care in Last Year

Reasons	2022	2023
Cost of Healthcare	17%	60%
Preferred Provider Unavailable	56%	16%
Transportation Problems	43%	8%
Work or Family Responsibilities	14%	8%
English Language Barriers	43%	8%

### Services Utilized

In 2022, the three most commonly used services were glucose and cholesterol testing, flu vaccination, and blood pressure check, respectively. Attendees were most interested in receiving vision and glaucoma screenings, followed by general screenings, and cholesterol screenings. Dental screenings were the most in-demand service that was not available at the fair followed by women’s health services (i.e., mammograms, breast exams, gynecological exams).

In 2023, the three most commonly used services were glucose testing, blood pressure check, and cholesterol testing, respectively. Attendees were most interested in receiving dental screenings, followed by vision and glaucoma screenings, and general screenings. Attendees were most interested in increased availability of dental screenings at subsequent fairs.

### Volunteers

In 2022, there were 223 volunteers consisting of medical students, physician assistant students, physical therapy students, occupational therapy students, physician assistant students, law students, residents, attendings, and community partners. Out of the volunteers, 24 served as Spanish interpreters.

In 2023, there were 274 volunteers consisting of medical students, dental students, physical therapy students, occupational therapy students, physician assistant students, residents, attendings, and community partners. Out of the volunteers, 41 of them served as Spanish interpreters.

## Discussion

STHF was placed in hiatus for two years due to the pandemic and reinstated in 2022, with potential changes to the healthcare needs of the underserved population in Winston-Salem since the 2019 STHF. When comparing 2022 STHF with 2023 STHF, there were many similarities with the attendee demographics. In both years, the Hispanic or Latino population made up the majority of the attendees, with an increase of 13% between the years. As far as race, there were similar numbers (57% and 56%) for those that identified as other races. Given the fact that the Hispanic or Latino population makes up the third largest population in Forsyth County, they make up an unproportionally high number of the attendees at the fair, further confirming the potential healthcare disparities, including lack of insurance or primary care physicians that exist within the community [4]. There was an increase in the number of return attendees from 2022 to 2023.

Of note, prior to 2022, the highest recorded fair attendance was 213 individuals in 2019 with 70% Hispanic or Latino individuals, further demonstrating an increase demand and need for free health services. The change in attendee demographic might be attributable to difference in advertisement methods for the fair or as a result of the COVID-19 pandemic, in conjunction with STHF going on hiatus in 2020 that further widened the gap in healthcare access and socioeconomic difficulties in this population. Many individuals in North Carolina, most notably in western North Carolina, report the need to delay seeking care during the pandemic as a result of prioritizing paying utility bills over health needs or fear of being out in public [10]. The federal poverty thresholds are updated each year by the United States Census Bureau; with current guidelines being $15,060 for one individual or $31,200 for a household of four [11]. Over 50% of our fair attendees in both 2022 and 2023 earned less than $15,000 annually for their total household. According to the North Carolina State Health Improvement Plan, in 2021, 23.4% of the Hispanic population lived below the federal poverty line [12]. Thus, Winston-Salem has a disproportionality higher number of individuals living below the poverty line when compared to state averages. This further demonstrates the need to increase free health services offered in this county.

Dental screenings and retina/glaucoma screenings have consistently been the most desired services at the fair, which shows the need for increased access to these resources. Retina and glaucoma screenings were first introduced to the fair in 2022 and continued in 2023, but there continues to be a need for it within the community. During both years, the attendees have waited hours for the services, causing many individuals to miss out on this service. This demonstrates the paucity of free eye screening services within the community. Dental screenings were offered for the first time in 2023 in a partnership with dental students in Chapel Hill.

STHF exists as a source of primary care access for the Winston-Salem community. Future improvements for STHF include the continued expansion of dental service availability and working to obtain equipment to increase the number of individuals that can be screened. As an expansion on STHF efforts, there will be an expansion on the retina/glaucoma screenings provided at the fair with several vision screening events held throughout the year in an effort to screen everyone that requires the services.

STHF has been shown to be a viable and important health screening event in Winston-Salem and surrounding areas. The prevalence of healthcare disparities and lack of healthcare maintenance across the Winston-Salem population that was exacerbated during the pandemic correlates with the increased attendance at the fair. The completely student-run event provides students with valuable exposure in the population they are studying to treat and provides the community with healthcare access that attendees may not be able to acquire otherwise.

## Data Availability

No datasets were generated or analysed during the current study.
